# T cell populations are negatively correlated with natural killer and macrophage cell populations in aspirate samples of peripheral lymphadenopathies

**DOI:** 10.1080/21505594.2026.2624191

**Published:** 2026-01-28

**Authors:** Philip J. Moos, Allison F. Carey, Jacklyn Joseph, Stephanie Kialo, Joe Norrie, Julie M. Moyareke, Anthony Amof, Hans Nogua, Albebson L. Lim, Louis R. Barrows

**Affiliations:** a Department of Pharmacology and Toxicology, University of Utah, Salt Lake City, UT, USA; b Department of Pathology, University of Utah, Salt Lake City, UT, USA; c Coordinator of Pathology Services, Port Moresby General Hospital, Boroko, Papua New Guinea; d Papua New Guinea National Department of Health, PMGH, Division of Pathology, School of Medicine and Health Sciences, University of Papua New Guinea and Central Public Health Laboratory, Boroko, Papua New Guinea; e Department of Medicinal Chemistry, University of Utah, Salt Lake City, UT, USA

**Keywords:** Mycobacterium tuberculosis, lymphadenopathy, single cell RNA sequencing

## Abstract

We employed single-cell RNA sequencing (scRNA-seq) of fine needle aspirates (FNAs) to describe the cells and communication networks characterizing granulomatous lymph nodes of TB patients. We uniformly identified several cell types known to characterize granulomas. Overall, we found the T cell cluster to be the most abundant. Other cell clusters that were uniformly detected, but that varied in abundance amongst the individual patient samples, were the B cell, plasma cell and macrophage/dendritic and NK cell clusters. When we combined all our scRNA-seq data from our current 19 patients, we distinguished T, B, macrophage, dendritic and plasma cell subclusters. The sizes of these subclusters also varied dramatically amongst the individual patients. In comparing FNA composition we noted trends in which T cell populations were negatively correlated with NK cell populations and with macrophage/dendritic cell populations. In addition, we discovered that the scRNA-seq pipeline detects Mtb RNA transcripts and associates them with their host cell’s transcriptome, thus identifying individual infected cells. The number of infected cells also varies in abundance amongst the patient samples. CellChat analysis identified predominating signaling pathways amongst the cells comprising the various granulomatous lymph nodes, identifying several pathways involved in immune cell maturation, migration and adhesion.

## Summary

The research conducted describes the cellular composition and communication networks within granulomatous lymph nodes of tuberculosis (TB) patients, employing a single-cell RNA sequencing (scRNA-seq) approach. By analyzing individual patient samples and clustering cells based on their transcriptome similarities, the study reveals several consistent cell types described to be present in both human and non-human primate granulomas. Notably, T cells emerge as abundant in most samples. Additionally, variations in the abundance of B cells, plasma cells, macrophages/dendritic cells, and Natural Killer (NK) cells among patient samples are observed. Pooling scRNA-seq data from 19 patients enabled the identification of T, macrophage, dendritic, and plasma cell subclusters, each displaying distinct signaling activities. A trend identified in the sample set inversely correlates T cell abundance with that of NK cells. Also detected was a negative correlation between T cell populations and cells of monocytic and macrophage lineages. Moreover, the study uncovers a surprising capability of the scRNA-seq pipeline to detect Mtb RNA transcripts within host cells, providing insights into individual infected cells and Mtb burden. CellChat analysis reveals predominant signaling pathways within granulomas, highlighting interactions between stromal/endothelial cells and the immune cell components. Selective communication pathways involving molecules such as MHC-I, MHC-II, MIF, CD45, galectin, APP, CD99, LCK, ICAM, NEGR, NCAM, CD6, TGFβ, PECAM and prostaglandins are identified, shedding light on the intricate interplay of cells within granulomatous lymph nodes during TB infection.

## Introduction

Lymphadenopathy refers to the swelling of lymph nodes that can be secondary to bacterial, viral, or fungal infections, autoimmune disease, and malignancy [1,2]. Once malignancy is ruled out, the association of the lymphadenopathy with a medical history and symptoms consistent with tuberculosis (TB) is a key diagnostic consideration in TB-endemic areas. Granulomatous lymphadenopathy caused by *Mycobacterium tuberculosis* (Mtb) is a signature feature of tuberculosis. Such granulomas are composed of immune and nonimmune cells that are thought to mount an orchestrated pro- and anti-inflammatory response to the bacterium [3–12]. Long deemed protective to the host, the granuloma is now understood to benefit the bacterium in some ways and represent a site of critical host–pathogen interactions that drive infection outcomes [12]. We present here the first single cell transcriptomic analysis of human peripheral tuberculosis-associated granulomatous lymphadenopathies. This analysis highlights new perspectives gained by single cell analysis [13].

TB remains a major global health emergency, causing over 1.5 million deaths in 2022 [6,14–16]. In low-resource settings, the diagnosis of TB is often based on history and symptomology, supported by acid-fast Ziehl-Neelsen staining microscopy of sputum or biopsy smear, and GeneXpert molecular testing when available [17,18]. It is estimated that 50% or less of all active TB cases are identified by microscopy in such resource limited settings [19–21]. At the study site, the TB clinic of Port Moresby General Hospital, Papua New Guinea, it is standard protocol to take FNAs of peripheral lymph nodes greater than 0.8 cm in diameter in presenting TB patients, in order to obtain samples for acid-fast microscopy and GeneXpert analysis.

We obtained FNA samples from such TB patients (S-Table 1) and report here the single-cell RNA sequencing (scRNA-seq) characterization of the cell types detected and analysis of their communication networks. When mapping cells from individual patient samples, clustered based on their transcriptome similarities, we uniformly identify several cell types that are known to comprise granulomatous lymphadenopathies in humans and non-human primates (NHPs) [22,23]. We find the T cell cluster to be one of the most consistent and abundant. Other cell clusters that are uniformly detected, but which vary dramatically in abundance amongst the individual patient samples, were the Natural Killer (NK) cell, B cell, macrophage/dendritic cells, and plasma cell clusters. When we combined all the scRNA-seq data from our current 19 patients (in order to add power to cell cluster identification in patient samples with fewer cells or of poorer depth of coverage), we distinguish T, B, macrophage, dendritic cell and plasma cell subclusters, each with distinct signaling activities. The sizes of these subclusters also varies dramatically amongst the individual patients suggesting that the predominance of their respective immune signaling pathways relevant to bacterial control may vary proportionately. In analyzing the cellular composition of our samples with sufficient depth of coverage to cluster NK cells separately from T cells, we defined several statistically significant trends in the FNA cellular composition. The most significant being that as the percentage of NK cells increases, T cell numbers decrease. A second trend that was identified suggests an increase in macrophage and dendritic cells as NK cells increase and T cell percentages decrease.

**Table 1.** Selectively expressed cell marker genes used for cluster-cell-type identification, with other tools.

Selectively expressed cell marker transcripts			
Name	Cell type	Name	Cell type
CD4	T helper cells	LILRA4	Dendrocytes
CD3D	T cells	LAMP5	Dendrocytes
CD8A	T cytotoxic cells	LRRC26	Dendrocytes
TIGIT	T cells	FDCSP	Germinal dendrocytic cells
FOXP3	T repressor cells	ACSL1	Foam cell
IL7RA	T cells	LTA4H	Caseous
GZMA	T cytotoxic cells, NK cells	RNASE1	Macrophages, endothelial
TBX21	Th1 cells, NK cells	COL3A1	Endothelial
NKG7	NK cells	COL4A1	Endothelial
CX3CR1	NK cells	CD31	Endothelial, monocytes, adipocytes
FUT4	Neutrophils	EMP1	Endothelial, keratinocytes, adipocytes
CD14	Macrophages	EPAS1	Endothelial, smooth muscle, adipocytes
CD163	Macrophages	CD34	Endothelial, smooth muscle, adipocytes, glandular cells
APOC1	Macrophages	CD40	B cells
CSF1R	Macrophages	CD79A	B cells
CD68	Macrophages	CD24	B cells
CPVL	Macrophages	MS4A1	B cells
LYZ	Macrophages	CD19	B cells, plasma cells
C1QB	Macrophages	IGHG1	Plasma cells
LILRA1	Macrophages	JCHAIN	Plasma cells
LILRA5	Macrophages	HBA1	RBC
CLEC4C	Dendrocytes	HBB	RBC
SCT	Dendrocytes	HBG2	RBC

In a surprising result, we also discovered that the scRNA-seq pipeline [24,25], designed for quantification of human mRNA, also detects Mtb RNA transcripts and associates them with their infected host cell’s transcriptome, thus identifying individual infected cells. The numbers of Mtb reads and infected cells also vary dramatically in abundance amongst the patient samples. We postulate that the number of detected bacterial transcript reads provides a measure of Mtb burden, as does the number of Mtb-infected cells. In comparing statistically differentially expressed transcript features revealed in heat maps, it was noted that MALAT1 [26,27], a long, non-coding RNA is significantly down-regulated in Mtb-infected cells, among other changes.

CellChat analysis revealed the predominating signaling pathways within granulomas, highlighting interactions between stromal/endothelial cells and the immune cell components. Selective communication pathways involving molecules including MHC-I, MHC-II, MIF, CD45, galectin, APP, CD99, LCK, ICAM, NEGR, NCAM, CD6, TGFβ, PECAM and prostaglandins were identified, shedding light on the intricate cell-cell interplay within granulomatous lymph nodes during TB infection. Thus, scRNA-seq analysis of this first of-its kind set of human TB FNA samples has allowed us to identify cell-cell communication ligands and receptors likely to play important roles in the functioning of the human peripheral lymph node granuloma. Further, it has identified trends in granuloma composition that may correlate with their relative inflammatory status. This initial single cell analysis of 19 human FNAs is consistent with literature suggesting a great deal of variation in cellular composition and bacterial load in FNA samples from TB patients with peripheral lymphadenopathy.

## Results

### Determination of FNA cellular composition

**Figure 1.**
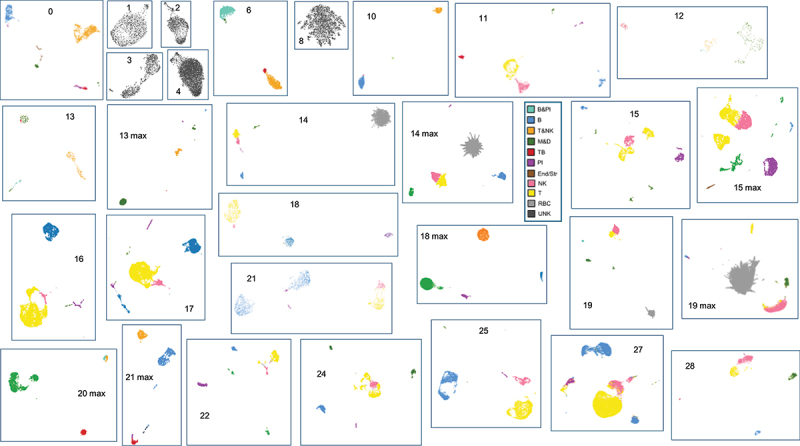
Individual FNA UMAPs and cell cluster identifications. CellRanger indexed BAM files as cloupe.Cloupe files were visualized using the 10XGenomics Loupe Browser 7.0.1. Reads were mapped to human (hg38), H37Rv Mtb (NC_00096.3:1) and HIV-1 (NC_001802.1) genomes and dimension reduction accomplished using the Loupe Browser designating 30 PCAs and clustering to the nearest 10 cells. FNA number shows in each panel. Cell types constituting the recognized clusters for each FNA were identified by the differential expression of selective marker transcripts (Table 1). Some FNAs with fewer numbers of cells were run through the entire 10XGenomics pipeline a second time (designated max) starting with the maximum possible volume of cells for analysis. For FNA 10, the RNA had degraded and yielded a poor quality max library during the re-run, and is not included here. For FNA 20, the initial library was too small to include as well. Significant erythrocyte contamination is apparent in FNA 14 and 14 max, and FNA 19 and 19 max. For the 10 most discriminating UMAPS, T and NK cells could be enumerated in separate clusters. The other FNA samples, with less resolution, showed intermingled T and NK or intermingled B and plasma cells (clusters T&NK and B&Pl, respectively), which were enumerated as such to calculate the FNA cellular compositions shown in Figure 2.

In 2016, we began a collaboration with Dr. Evelyn Lavu (decd.) and colleagues at the Port Moresby General Hospital (POMGH) Tuberculosis Clinic, Papua New Guinea, to obtain an MRC-approved (IRB) protocol that allowed us to attempt scRNA-seq analysis on human FNA samples from peripheral nodal granulomas of tuberculosis patients. At the POMGH, TB patients referred to the clinic are assessed based on presentation, symptoms and history. Fine needle aspiration (FNA) is the established protocol to assist diagnosis of patients exhibiting accessible lymph node granulomas exceeding 0.8 cm diameter. For patients agreeing to this assessment, three fine needle aspirate passes of the granuloma are harvested for acid-fast microscopy and for GeneXpert analysis [17,18]. Our protocol provided us one of these aspirate samples, which was preserved on-site in methanol for sterilization, transportation and subsequent re-hydration. After several rounds of pre-preservation sample clean-up optimization, we have accomplished scRNA-seq analysis of FNA samples from 19 individual patients presented here (Figure 1). While not all of our patients confirmed positive for TB using microscopy or GeneXpert analysis, all were diagnosed as suffering from active TB and were prescribed antibiotic therapy.

**Figure 2.**
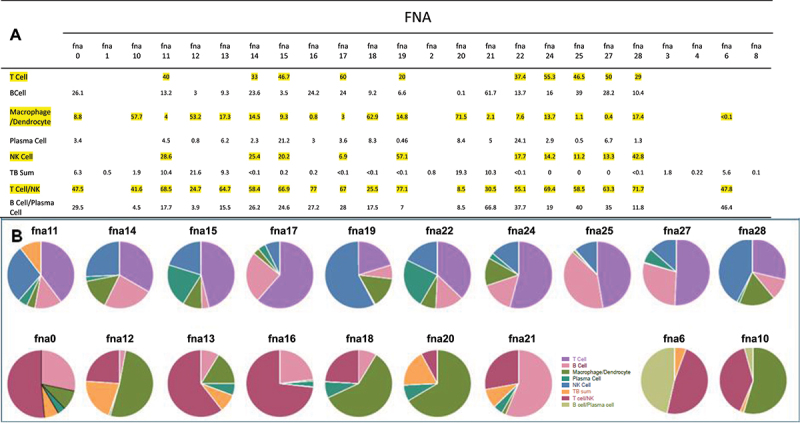
Cell composition of individual FNA clusters. Panel (A) a matrix of identified cell cluster percentages making up the individual FNAs as resolved by the Loupe browser and identified by differential expression of marker transcripts (from Figure 1). Raw cell count totals are presented in (fig. S-1). Panel (B) pie charts of FNA composition. Upper panel of pie charts shows FNAs for which NK cell clusters could be quantified separately from T cells (FNA 11, 14, 15, 17, 19, 22, 24, 25, 27 and 28). Cluster identities were later confirmed by algorithm driven cell clustering in the combined data set. Lower panel of pie charts shows the FNAs with intermingled T and NK cell clusters (FNA 0, 12, 13, 16, 18, 20 and 21), and those also with intermingled B and plasma cell clusters (FNA 6 and 10). FNAs 1, 2, 3, 4 and 8, while passing UMAP and Seurat QC settings, displayed low coverage of PC transcripts and failed to yield clustering of component cells, although each had detectable populations of MTB infected cells. These FNAs were not included when calculating FNA cellular composition.

In 2019 we began to obtain de-identified FNA samples from permission-granting patients. Only one sample from 2019 yielded a library of sufficient quality for sequencing (FNA 0). From 2022, using improved protocols we obtained libraries from six FNA samples; however, most of these (FNAs 1, 2, 3, 4 and 8; Figure 2) had high numbers of cells that were essentially unidentifiable when analyzed individually because of the lack of discriminating transcripts. Even though all these FNA libraries passed rigorous QC settings, the coverage was too low to allow discrimination amongst cell types and so were not included in the following analysis. With further improvement of our pre-preservation protocol (see Methods), we had better success with FNAs collected in 2023, obtaining high-quality libraries and sufficient numbers of cells from most of the FNAs 10 through 28.

After rehydration and processing through the 10X Genomics pipeline for scRNA-seq and Illumina sequencing, the transcriptomes were assembled, annotated and data visualized using Cell Ranger and Seurat v4/tSne or UMAP analyses and dimension reduction tools (see Materials and Methods) to yield the 10X Genomics Loupe Browser 7.0.1 UMAPs shown (Figure 1). Because of the low cell numbers detected in the initial analyses, some of the FNAs were re-run through the entire 10X Genomics pipeline at the maximum cell volume (designated max). When both the initial and the max re-runs yielded libraries of sufficient quantity and quality, the data were combined. The differential expression of selective cellular transcripts (Table 1), obtained from the literature and our Loupe Browser 7.0.1 heat map analyses (selectivity vetted using the Human Protein Atlas website [28]) was used to identify and quantify the cell types detected in the FNA samples in Figure 1 in order to determine their cellular make up (Figure 2). CellMarker 2.0 [29], SingleR 1.12.1 [30], Azimuth [31] and CellTypist [32] cell predicting analyses of the combined data set (below), were also used to confirm the cell types as shown (Figure 1 and 2). While each patient sample was different, with FNA volumes as low as ~100 µL, the numbers of cells recovered after preservation in methanol ranged narrowly, from approximately 7X10^6^/mL to 6X10^7^/mL. This provided an excess of cells for scRNA-seq analysis and allowed re-running specified samples.

When analyzed individually using the Loupe browser or Seurat dimension reduction tools, most of the FNA samples yielded UMAPs with a limited numbers of clusters that included most of the component cells, usually easily attributable to given cell types based on marker transcripts, (Figure 1). Most commonly identifiable was a T cell cluster (CD3D, IL7R, CD8A, etc., positive). The T cell cluster was often intermingled with NKG7 (natural killer) cells, depending on the number of cells and the coverage of the library (Figure 2). When this was the case the cluster was designated T&NK, rather than clearly definable as individual T and NK clusters. Other clusters routinely detected included a B cell cluster (MS4A1, CD40, CD79A, etc., positive cells), a plasma cell cluster (JCHAIN and IGHG1 positive), a macrophage/dendritic cell cluster (CD14, CD68, CD163, CPVL, LYZ, CEC4C, LILRA4, etc., positive), and a TB positive cluster (Mtb rrs and/or rrl transcript positive, which encode ribosomal RNAs). It was clear that while the T cell cluster was uniformly detected as one of the most abundant harvested from every patient, the other cell type clusters varied dramatically (Figure 2). As mentioned, in some FNAs with smaller libraries, or less coverage, individual UMAPs could not distinguish meaningfully amongst the detected T and NK, or B and plasma cells. Cells expressing T or B cell subtype, or macrophage/dendritic cell differential subtype markers, were routinely intermingled into the single the T, B or macrophage/dendritic cell Loupe Browser UMAP clusters, respectively. Cell numbers varied from a low of 219 for FNA 12 to a maximum of 26,804 for FNA 27 (Fig. S-1). Only infrequently did we detect clusters of cells that are identifiable as something other than T, B, plasma, macrophage/monocyte or dendritic cell types (e.g. cells expressing “selective markers” for putative erythroblasts or serosal and stromal cells). We did not detect a distinct neutrophil cluster, probably because they are known to be transcriptionally inactive and not easily harvested in significant number by fine needle aspiration [33]. SingleR analysis of the combined data set suggests they cluster with other phagocytes (Fig. S-2, S-3).

We first sought to define the cellular composition of the 19 FNA samples and to look for any trends in that data. Several approaches were taken to quantify cell clusters generated in the Loupe Browser UMAPS. Initially, barcodes of cells displaying a selective marker were quantified and summed to 100% for a given UMAP. However, with the relatively low coverage we found in some of our samples the majority of cells went uncounted. Furthermore, it was uncertain whether a given marker for T cells would yield barcode numbers as efficiently as a given marker for B cells, etc. As an alternative, the totals of all the cells comprising the identified cell type clusters for each sample UMAP (Figure 1) were determined individually, using K means differentiation and the Loupe Browser lasso tool to quantify the cells comprising a given cell type cluster. These cells were then summed to determine the percentage contribution of each cluster to the cell total in a given UMAP (Figure 2). It was these percentages that were used to generate the pie charts shown in Figure 2. These percentages were later confirmed (below) using algorithmically determined cluster percentages from the combined data set.

**Figure 3.**
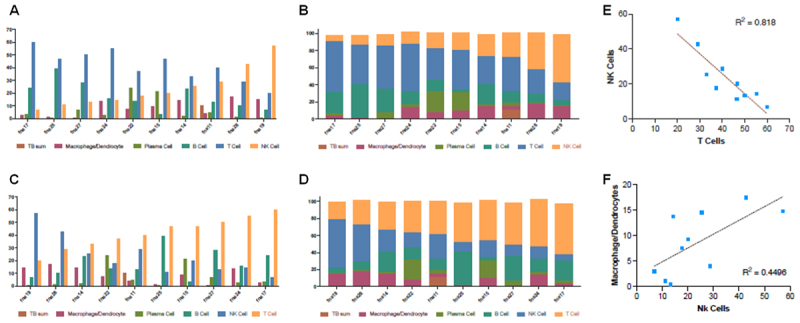
Trends in cellular composition of FNA samples. the percentage of cell types making up given FNAs (from Figure 2) were plotted as histograms or stacked bar charts to identify obvious trends (panels A and B show others cell percentages versus increasing NK cells; and panels C and D show others versus increasing T cells). Trends suggested in panels A-D were then evaluated by fitting trend lines to scatter plots of the data (panels E and F). Significant trends were identified suggesting an inverse relationship between the numbers of NK cells and T cells, and a positive relationship between numbers of NK cells and macrophage and dendritic cell cluster.

Initial analyses tracking cellular composition versus increasing Mtb-infected cells did not reveal any significant trends. Neither did other analyses tracking FNA composition versus increasing numbers of the other comprising cell types if samples with the combined T&NK cluster percentages were included (e.g. T&NK clusters in Figure 2). However, when we focused on the 10 most discriminating FNAs, in which a T cell cluster could be distinguished from the NK cell cluster, a trend was identified in which increasing NK cell percentage was inversely related to the number of T cells (Figure 3). A second trend was identified, in which there is a suggestion that the macrophage/dendritic cell percentages increases proportionately with NK cell number in the FNA (or inversely with T cell number). To further assess the inverse relationship between T cells and NK cells, as they comprise these FNA samples, we tracked our selective T cell marker barcodes versus increasing NK cell barcode numbers, but consistent trends were not identified comparing between the different cell predictor strategies and tools used.

### Cellular communications indicated by the combined data set

**Figure 4.**
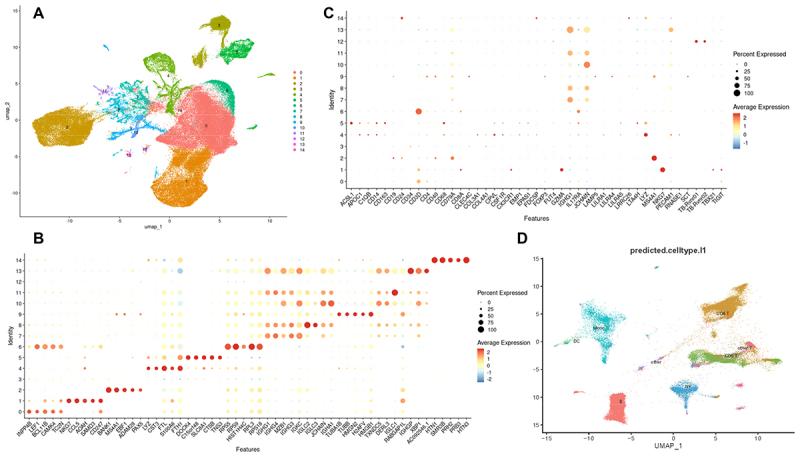
Combined data set cluster identification combination of all approximately 108,000 cells from our combined 19 FNAs (initial and max reruns combined) in Seurat and SingleR analysis designating a resolution of 0.1, yielded the UMAP shown (panel A). The top 5 most differentially expressed genes identified in the 15 cell clusters using this analysis are shown in panel B and expression of our selective marker genes shown in panel C. Our marker gene set was used in combination with SingleR (fig. S-3) to predict cell types constituting the 15 clusters. Azimuth cell reference-based mapping pipeline (panel D) analyzed the same combined data set and was also successful at clustering confirming the identify cell types making up the clusters identified in the combined data set. Going forward, the Azimuth and Seurat/SingleR pipelines for cell annotation were employed as the primary reference sets to confirm the Loupe Browser data.

**Figure 5.**
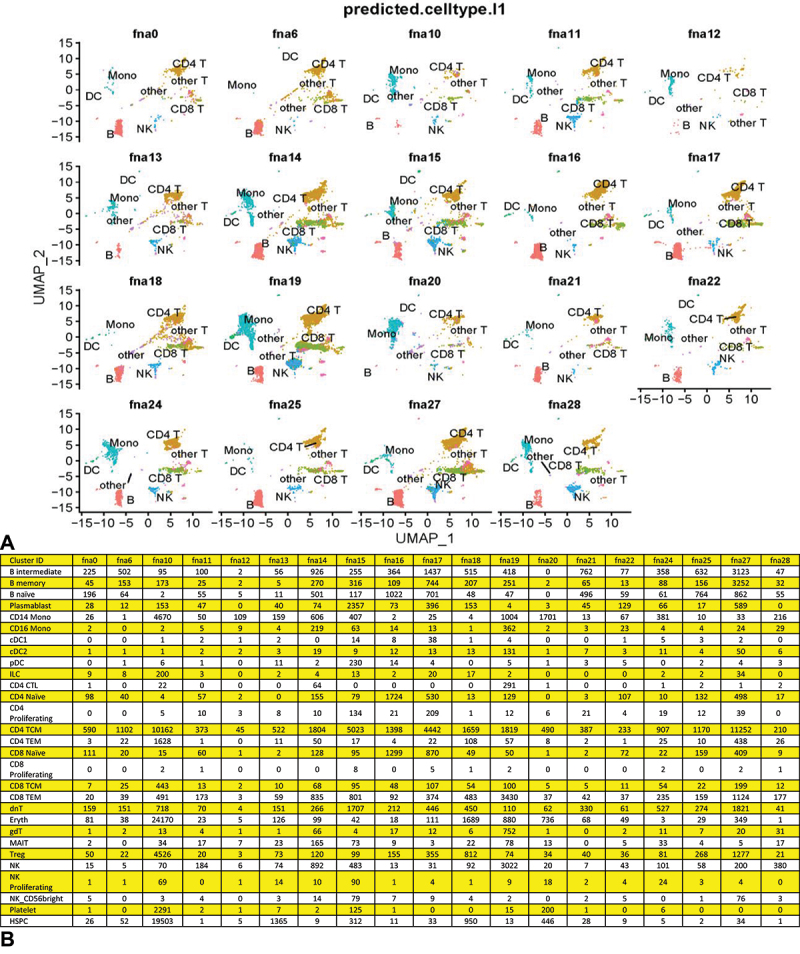
Composition of individual FNAs determined from the combined data Set. (panel A) the individual FNA UMAPS extracted from the combined Azimuth UMAP (from Figure 4D, S-5) show that the strategy to combine all our patient samples in order to increase the precision with which cell types could identified was successful. The percentages of parental cell types was consistent with the percentages assigned in Figure 2. A matrix of the percentages/100 of each of the identified cell clusters at Azimuth resolution.L2 is shown in panel B. HSPC cells identify as stromal/endothelial.

To increase our power, and to attain greater precision in cell type identification, we decided to combine all the patient data into one UMAP in order to gain power for algorithmic cell type identification (Figure 4) and (Fig. S-3). This increased number would also empower meaningful description of cell-cell communication networks of cells characterizing the combined FNA data set. While requiring greatly increased computing power, combining the approximately 108,000 cell transcriptomes from our 19 patient FNAs did provide increased confidence in the ability to visualize the distribution of cell types amongst our various patient samples (Figure 5). Attempts to assign identities to cells making up Cluster 3 from the 10X Genomics Seurat clustering tool using SingleR or our discriminating marker set was difficult because it was comprised of relatively transcriptionally inactive cells. This was not an issue using the Azimuth cell reference-based mapping pipeline [31] (Figure 4; S-5), possibly due to Azimuth having a better matched single-cell RNA reference transcriptome. The reference transcriptome we employed for Azimuth analysis was the PBMC data set [31] because it had the highest degree of statistical agreement with the transcriptomes in cell types from our data set. For instance, we found it to match better than the human tonsil V2 data set option. We also compared analysis with the CellTypist cell predictor tool [32] but found it to identify cell types not reported as contained in the lymph node (e.g. Temra T cells) and so for simplicity’s sake we limited our study to the SingleR and Azimuth annotation tools.

As shown in Figure 5, the composition of individual FNAs could be extracted from the Combined Data Set. As an independent, fully algorithm-driven dimension reduction-clustering results, we asked if the ratios of the major T, B, macrophage/dendritic cell, NK, plasma and TB cell cluster percentages from the individual extracted FNAs agreed with those determined by the individual Loupe analyses shown in Figure 2. When the respective cell type sub-clusters from the combined data set analyses were summed and the percentages of the total cell number from the FNAs determined, there was close agreement of the FNA compositions extracted from the combined data set using either the SingleR or Azimuth cell reference-based mapping pipeline tools (Figure 5, S-1, S-3) when compared to each other or when compared to the FNA compositions determined by the individual Loupe analyses (Figure 2). In addition, when the trends correlating increasing NK cell percentages inversely with T cell percentages and positively versus macrophage/dendritic cell percentages were tested, they were confirmed with R^2^ values of 0.9, 0.8 and 0.71 for NK vs. T, T vs. MMD and NK vs MMD, respectively when using the combined SingleR UMAP data set; and 0.6, 0.6 and 0.6 respectively when using the combined Azimuth UMAP data set (Fig. S-4).

**Figure 6.**
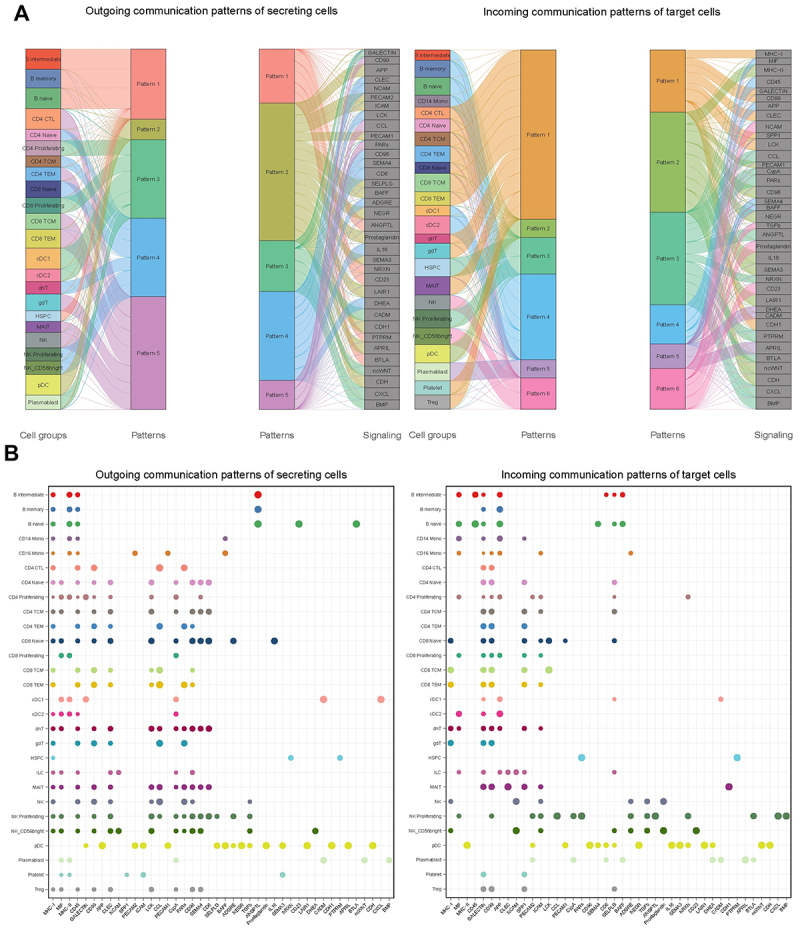
CellChat signaling interactions for combined FNA DATA set. The patterns of signaling interactions between secreting and receiving cells in the combined data set cell clusters identified using Azimuth analysisat.L2 resolution and their relative patterns are shown in panel A. While the signaling interactions are many, the predominant signaling pathways identified are MHC-I, MHC-II, MIF, CD45, CD99, galectin, APP CD99 and so on. Panel B shows the relative strengths of “outgoing” and “Incoming” communication ligands and receptors for the individual cell clusters identified using Azimuth analysis at.L2 resolution. These signaling interactions are likely interactions of cells comprising a granulomatous lymph node.

The CellChat tool (Figure 6) was used to predict cell communication ligands and receptors expressed in the combined data set clusters. CellChat quantifies the signaling communication probability between two cell groups using a simplified mass action-based model, which incorporates the core interaction between ligands and receptors with multi-subunit structure along with modulation by cofactors [34]. Cell-Cell communications of the various T, B, macrophage/dendritic, NK and plasma cell subclusters suggests that Seurat and Azimuth UMAP clustering of the combined data set did in fact distinguish amongst cell-type subsets based to some extent on their signaling status. When evaluated, it was found that these same “Sender and Receiver” ligands and receptors transcript markers, designated as selective for a particular cell-type subset from the combined data, mapped to the appropriate respective umbrella-cell-type cluster in the individual Loupe Browser UMAPS. Thus, monocytic and dendritic cluster cell-specific ligand transcripts from the combined data set, mapped to the single macrophage/dendritic cell cluster identified in the Loupe Browser UMAP of individual FNA analyses, and so on. This again supported the consistency between our various approaches to the FNA analyses.

At resolution.l1, Azimuth analysis identified MHC-I, MHC-II, MIF, CD45, galectin, APP, CD99, LCK, ICAM, NEGR, NCAM, CD6, TGFβ, PECAM and prostaglandins as the top signaling interactions. CellChat data obtained using the Azimuth.l2 (Fig. S-5) level of resolution predicted cell identities is presented in Figure 6. The resolution.l2-predicted interactions suggest more precise interactions between significant populations of cell subtypes, some of which are quite selective. For instance, naive B cells are predicted to be the only secreting cell subtype signaling via CD23 and BYLA, the plasmacytoid dendritic cells appear to be active in autocrine signaling via APRIL and CDH pathways, etc. All these same pathways of intercellular communication were also identified when the CellChat was run using the combined Seurat SingleR UMAP clusters (S-2), showing the consistency of the analysis. Many of the identified communication interactions have to do with immune cell activation (e.g. MIF, MHC-1&II, CD45, etc.) whereas a significant portion of the other interactions seem to affect cellular migration and adhesion (e.g. Galectin, PECAM, ICAM, CD99, etc.). All of these signaling interactions are intuitively consistent with interactions expected of cells comprising a granulomatous lymph node.

### Status of Mtb-infected cells

**Figure 7.**
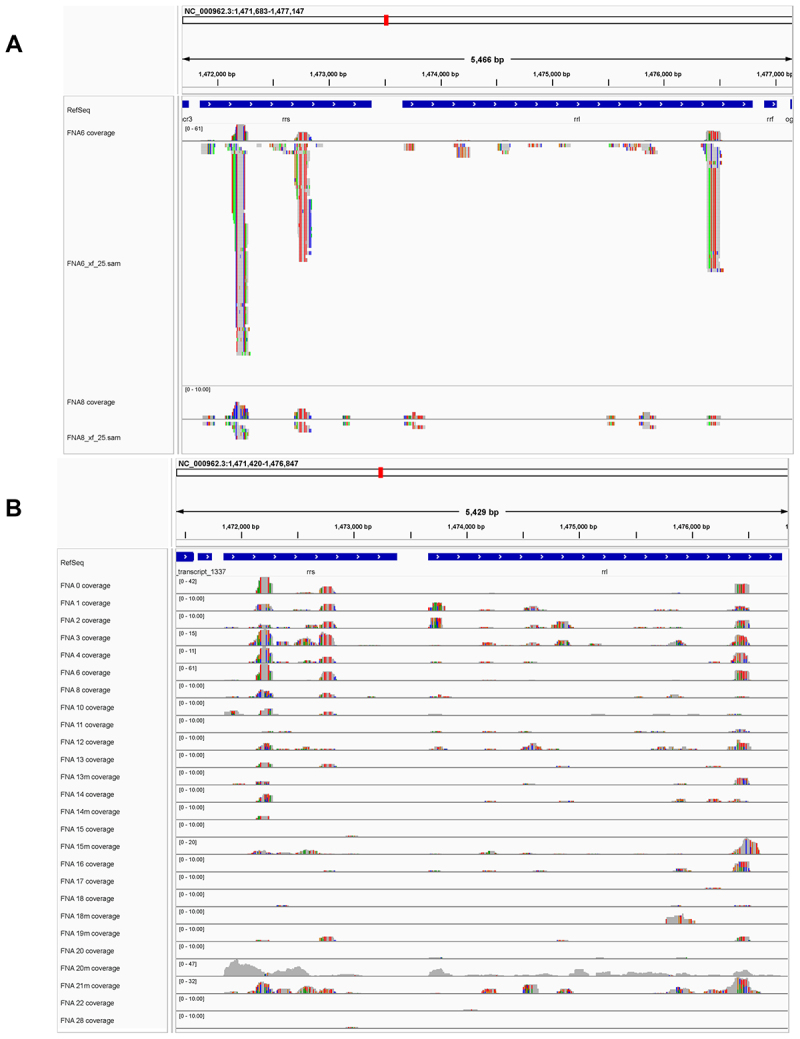
Coverage of rrs and rrl genes by Mtb reads detected in FNAs. No bacterial sequences were detected in FNA 22, 24, 25, 27. IGV alignment of Mtb reads associated with host cell UMIs from FNA 6 and FNA 8, respectively (panel A), illustrates the variation between FNA 6 and FNA 8 in the numbers of transcript reads mapping to the small (rrs) and large (rrl) ribosomal RNA genes in the reference Mtb H37Rv genome. Multiple reads of identical length, starting position and directionality, usually in pairs or sets of three, are interpreted as arising from a single RNA source which we hypothesize was then amplified during library generation. panel B shows the coverage of rrs and rrl genes in reads detected in the complete FNA set. Sequence variations versus the reference H37Rv genome are show in colors: G (brown), a (green), C (blue) and T (red).

**Figure 8.**
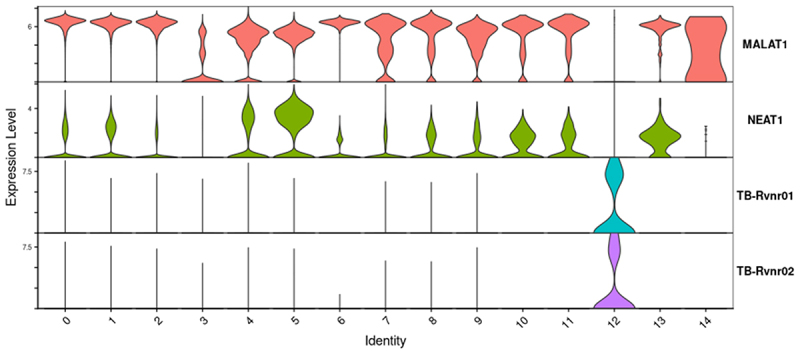
Expression of MALAT 1 in 10X Genomics pipeline combined data Set. Relative MALAT1 and NEAT1 expression in the 15 clusters identified in Figure 4. MALAT1 expression is significantly decreased in Mtb-infected cells (cells expressing Mtb rrs or rrl).

We hypothesize that quantification of Mtb reads (Figure 7), or of infected cells (e.g. Figure 2; infected cells can contain more than 1 detected Mtb.sam read), will correlate with Mtb burden. The detection of bacterial transcript reads appears to be a stochastic process, with most of the detected sequences being from either the rrs or rrl small and large ribosomal subunit genes (Rvnr01 and Rvnr02, respectively; Figure 7). This is likely due to the high level of ribosomal rRNA transcripts expressed in the Mtb cells [24,25]. However, it is possible that other features such as secondary structure and nucleotide modification could contribute to the spurious priming of these transcripts in the 10X Genomics pipeline, or perhaps even spurious priming with the UMI-leader sequence [24,25]. The 10X Genomics pipeline we used relies on poly-T primers to initiate reverse transcription. Clearly, some patient FNAs have relatively high numbers of Mtb reads (thousands) detected, while other patient samples with only a few or none (Figure 8). We had anticipated that trends of cellular composition in our FNA samples might correlate with increasing or decreasing numbers of Mtb infected cells in those samples. However, as reported above, we did not observe such trends in this data set when analyzed either using the individual FNA Loupe Browser or combined data set FNA compositions. Furthermore, when using CellChat to assess communications between cell type clusters, the TB-infected cluster (cluster 12 of the SingleR UMAP shown in Figure 2) appeared to be virtually silent.

When looking for selective transcript features of Mtb-infected cells in an *in vitro* macrophage/Mtb (THP-1-GFP-H37Ra) co-culture experiment [24,25], we noted that the long non-coding RNA, MALAT1, was routinely down-regulated in infected THP-1 cells. NEAT1 was found to be similarly down regulated, although less reliably in repeat experiments. Because of the possible immunomodulatory role of these long non-coding RNAs in viral infections [26,27] we asked if MALAT1 was downregulated in the TB-infected clusters of our patient FNAs. When assessing the 7 FNAs with highest overall numbers of Mtb-infected cells, we observed that in five of them (FNA 6, 11, 12, 13 and 21), MALAT1 was the most down-regulated transcript. In the other two FNAs assessed (0 and 20), MALAT1 was also significantly down regulated. The Expression of MALAT1 was then assessed in Mtb positive cells versus the other clusters identified in the Combined Data Set (Figure 8). This analysis confirmed down regulation of MALAT 1 in the Mtb infected cells across the complete data set. While many other transcriptional features vary amongst the FNA clusters, it is interesting that MALAT1 appears to stand out, perhaps suggesting a role in the bacterium-macrophage amour-haine relationship.

## Discussion

We present here the first scRNA-seq analysis of human fine needle aspirate samples from peripheral nodal lymphadenopathies associated with TB. We demonstrate such analysis is achievable in resource limited settings where higher incidences of tuberculosis often predominate. This proof-of-concept result provides new perspectives on the interactions between cells making up the presumed granulomatous lymphadenopathy. The data suggest that utilization of this innovative technology, with ongoing improvements in depth of library production and support of a state of the art microbiology laboratory, might provide important insights into patient status. Due to the work load at the tuberculosis clinic at POMGH, and the limited number of trained staff, there has been no opportunity as yet to obtain significant supporting pathology or microbiological follow up on the samples obtained there. There was confirmation of TB status of some of the FNAs by POMGH Pathology Laboratory microscopy of acid fast smears and/or GeneXpert analysis. Of our 24 de-identified FNA samples, 10 were confirmed as TB positive (FNAs 0, 4, 10, 13, 17, 18, 20, 21, 22 and 24; S-Table 1). Multiple publications have reported Zeihl–Neelsen confirmation of TB status to be between 25% and 60% in resource-limited settings [19–21]. GeneXpert confirmation in similar resource-limited settings is reported to be as high as 70%, when available [19,20]. Thus, lack of laboratory confirmation of TB status is only one component of patient evaluation and drug therapy is often begun based on patient history and presentation.

We found that one pass of fine needle aspiration yielded ~5 to 50 million cells for analysis in 1.25 mL 80% methanol, far in excess that is needed for scRNA-seq analysis. With experience we improved our pre-preservation protocol to include brief incubations with RBC lysis buffer and Accutase™ before methanol preservation, which yielded preserved FNA samples with suitable single cell suspensions after rehydration for the 10X Genomics scRNA-seq pipeline. We look forward to new innovations in preservation for single cell analysis which promise increased cell numbers and improved library quality and depth of coverage [35].

The cell types making up any one given FNA were assigned using our list of selective marker transcripts, in combination with SingleR [29], Azimuth [31] and CellTypist [32] cell annotation algorithms as well as other tools such as CellMarker_Augmented_2021 [29] and other tools [36,37]. Our list of selective gene transcripts was derived by laboriously vetting our own differential gene expression data, and from vetting many literature sources against our human nodal data set. Many putative literature markers for cell identification, (e.g. for Th or M subtype cells) were not found to be sufficiently selective or not sufficiently abundant in our data set to cluster those cells separately, although cells expressing those subset markers were easily quantifiable by barcode count. We included many more markers for monocyte/macrophage/dendritic cell types than the others because we found in our data set that often only limited numbers of the clustered putative monocytic/macrophage/dendritic cells expressed one or more of the selective markers, and often only one or two of the selective marker transcripts could be associated with that cluster. Furthermore, this varied from patient to patient. This was in contrast to the other clusters routinely identified in the individual UMAPS where CD3D and CD8A were found to be highly and selectively expressed in T cells, MS4A1 was found to be highly and selectively expressed in B cells, NKG7 was highly and selectively expressed in NK cells and JCHAIN and IGHG1 were highly and selectively expressed in plasma cells. These initial discriminating markers, in combination with the rest of the marker set, gave confidence to cell type identification, which was then tested for consistency with cell identifications generated with other tools. Further confirmation of the cell type identification (and quantification) was obtained later, when the cellular composition of the individual FNAs extracted from the combined data set were shown to agree with that of the individual Loupe Browser analyses. Additional agreement was also found when CellChat signaling data from the combined data set was correlated to the cells known to express those “Sender” or “Receiver” markers in the individual Loupe UMAPS. Thus, the individual Loupe UMAPS were consistent with the cell types known to express those signaling ligands or receptors.

Quantification of the given cell types comprising an individual FNA relied on the enumeration of cells in their respective clusters. We used K Means clustering tools to match Loupe Browser clusters to the cell types expressing genes on our feature list. These cluster populations were used to determine the percentage of total cells that cluster represented in a given FNA. On the occasions when K Means clustering divided or did not capture all the specifically clustered cells of a given type, the Loupe Browser Lasso tool was used to capture the cells as a single cluster. For about half the FNAs (Figure 2) the resolution of T cells and NK cells was good enough for confidence in assigning them to separate clusters, although these clusters often abutted or transitioned from one to the two dimensional representations. Unfortunately, while all the FNA libraries passed the same QC defaults in assembly and dimension reduction, the coverage of many of the libraries was insufficient to distinguish between closely transcriptionally related T and NK cell types. Nevertheless, repeated analyses yielded consistent cluster population estimations and were sufficient for the trend analyses presented in Figure 3 and S-4.

About half of the FNAs with small cell numbers or lower coverage yielded intermingled clusters of T and NK cells, and occasionally intermingled B and plasma cells. In these cases, the intermingled clusters were designated T&NK or B&Pl (Figure 1). When an individual cell type was particularly rare in a given FNA, such as in cases of FNAs with very few Mtb-infected cells, those cells often intermingled with various other named cell clusters and an estimation of their total count could only be obtained from the barcode count for the marker being used (e.g. TB_sum in the case of Mtb-infected cells). In FNAs with low Mtb counts, those Mtb-infected cells often associated with the plasma cell cluster in the individual Loupe Browser analyses, or less frequently with the macrophage/dendritic cell cluster. SingleR identified a separate Mtb-infected (cluster 12; Figure 4) in cells with high numbers of infected cells but Azimuth did not cluster those infected cells separately. During inspection of the individual Loupe Browser differential expression heat-maps, it was noted that Mtb-infected cells had the lowest relative MALAT1 expression of all the cell types, which suggests a role for MALAT1 down regulation in the bacterium-host cell interplay.

With confidence in cell type identification, we looked for trends or relationships between the cell types comprising the FNA samples (Figure 3). Extensive literature in human and NHP models suggests that cells making up the granuloma change with bacterial load and that this reflects mechanisms of bacterial control [38,39]. We initially looked for trends in cellular composition as we ranked FNAs according to increasing Mtb-infected cells, as determined by cluster analysis and Mtb barcodes. We did not find any significant trend in this limited data set. Only when we focused on samples in which we could distinguish NK cell clusters from T cell clusters did we identify significant cell composition trends. From these samples, we noted an inverse relationship between the two parental NK and T cells clusters. Another, less significant trend was noted as well, the increase of macrophage/dendritic cells with increasing NK cell percentage (or decreasing T cell percentage). These trends were confirmed with confidence (Fig. S-4) when the respective FNA cellular compositions were extracted from the combined data set (Figure 6) and recalculated using the SingleR cluster percentage numbers or with Azimuth cell reference-based mapping pipeline cell cluster percentage numbers although with less confidence. The identification of trends in cellular composition suggests the possibility that the maturation or “activity” of the infection in the lymph node will be reflected by the ratio of key cellular components (e.g. in NK/T cell ratios). Thus, the ratio of T cells to NK or macrophage lineage cells in FNAs may be convenient indicators of lymphadenopathy/granuloma status or maturity. This could be a useful clinical tool for determining patient status or prognosis [40,41]. Granulomatous tuberculosis lymph nodes are spatially organized structures, composed of a mixture of immune and nonimmune cells [4,5,40–42]. However, this three dimensional structure is lost to our analysis. However, the fact that we routinely obtain the spectrum of cell types described as foundational to granuloma formation, and that we detect Mtb infected cells in those samples, argues that we are indeed sampling the essential granuloma structure. Recent experiments using Visium HD [35] spatial transcriptomic analysis of formalin fixed paraffin embedded TB infected lymph nodes from three separate patients also shows a wide variation in the cellular composition, consistent with what we report here (experiments in analysis). We do not see several cell types reported in microscopic analysis of human or NHP studies in our FNAs [22,23]. We do not see significant numbers of neutrophils. As postulated earlier, we believe this to be in part due to their purported low levels of transcription and lack of selective markers and their possible sequestration to microabscesses [33,43]. Endothelial and stromal cells, also identified rarely in the combined data set, were only occasionally observed in the individual Loupe UMAPs, and never at high percentages.

In this initial report, we used CellChat to gain insight into the functionality of the cell types identified in the granulomatous lymph node FNAs, rather than focusing on differential gene expression. CellChat analysis was found to be insightful because it identified and ranked cell-cell communication ligands and receptors in the combined data sets. Many cytokines and chemokines have been identified as associated with Mtb infection [44,45]. Interestingly, IL10, IL17 and INFγ, TNFα or their receptors, were not among those we identified. These are cytokines reported by previous foundational studies as pivotal to nodal granuloma function in humans and non-human primates [22,23]. A study by Kathmandu and colleagues [45] reported elevated baseline levels of IL-13 and IL-10, with reduced levels of IL-4 and GM-CSF in lymph nodes of Mtb infected individuals. They also reported increased levels of IFNγ, TNFα, IL-2, IL-17F, IL-22, IL-1α and GM-CSF, with reduced levels of TGFβ in primary lymph node cultures challenged with Mtb antigens. A report of Zhang and coworkers [46], using bioinformatics analysis of lymph node tuberculosis identified CXCL9, CD36 and LEP as genes involved in the pathogenesis of lymph node tuberculosis.

Instead, MIF, MHC I, CD45, PECAM1&2, CD99, COLLAGEN, CYPA, ICAM, CD6, CAMD were among the most highly expressed communication systems identified here, although this varied depending on the data set (Seurat/SingleR or Azimuth UMAPS) and the resolution of discrimination for the identified cellular subtypes (Figure 6, S-3). Many of these mediators play key roles in macrophage activation or maturation (e.g. MIF and CYPA [47,48]), T cell activation and maturation (e.g. MHC I&II, CD45 [49,50]) and monocyte migration (e.g. TGFβ [51]). Many of the other identified ligands and receptors (Figure 6) participate in cell–cell interactions related to adhesion and maturation (e.g. PECAM1 and 2, Collagen, ICAM, CADM, CD99, etc. [52–56]). As mentioned before, when these signaling ligands or receptors were mapped in the individual Loupe UMAPS they all associated with their appropriate putative parent cell type, either “Sender” or “Receiver.” Almost all of these signaling interactions have immunomodulatory effects and are intuitively sensible, some having pro-inflammatory activities. It might be significant that our analyses are identifying cell signaling intrinsic to the granulomatous lymph node, rather than specifically relating the patient’s overall inflammatory status. We hypothesize that augmentation of interdiction of some of these key pathways could disrupt the structure or function of the granuloma and greatly affect that granuloma’s maturation or bacterial control.

Our data suggest that it is possible that the detection of Mtb transcripts associated with host cell UMIs might provide a measure of Mtb burden in the FNA and presumably, the granuloma. Due to a lack of laboratory resources in Papua New Guinea, it has not yet been possible to determine if the number of Mtb transcript reads, or if the number of detected infected cells, does indeed correlate with bacterial burden by colony forming unit (CFU) or chromosomal equivalent quantification (CEQ) analysis [57,58].

We anticipate that there are likely complications of co-infection with other viral, bacterial or fungal pathogens in FNA samples such as those studied here. This possibility might be addressable using meta-genomic approaches following of FNA aliquots. The complications introduced by HIV-1 co-infection might also be identified in studies that specifically include sufficient numbers of co-infected individuals. In our particular case, HIV-1 sequences indicative of HIV-1 co-infection were detected in two of the patient samples (FNA 0 and FNA 28). This information was relayed to the TB Clinic staff for follow up using established clinical protocols.

Overall, we believe this proof-of-concept study illuminates some of the potential insights that single cell analyses might provide into the structure and function of TB-associated lymphadenopathy. Our analysis identified cell composition and communication trends that possibly reflect lymphadenopathy status. The continuing rapid improvements in these single cell technologies promise additional new understandings of host–pathogen interactions in the future.

## Materials and methods

### FNA sampling

Fine needle aspiration samples were collected as follows: after granting permission, suspected pLNTB patients with nodal enlargement ≥0.8 cm underwent 2 to 3 passes into the affected lymph nodes with a 22-gauge needle. We have complied with all relevant ethical regulations concerning this work. De-identified patient data is shown in S-Table 1, each patient donated only 1 FNA. No more than one lymph node per patient was sampled. A new needle was used for each pass. One aspirate was washed directly into 9.5 mL ice-cold RPMI buffer containing 0.2% fetal bovine serum, in a heparinized tube and mixed gently.

Our 2019 protocol simply washed, pelleted, and re-suspended the cells in 80% methanol. Our 2022 protocol, added heparinized tubes, RBC lysis, and Acutase® digestion as in the final protocol, but with centrifugation and re-suspension between each step. This was quite lengthy and in the Papua New Guinea pathology lab there was no refrigerated centrifuge. So, while we had a number of samples pass QC (FNAs 1, 2, 3, 4, 6 and 8) they had poor coverage and more resembled nuclei in our experience than well-preserved whole cells. Our scRNA-seq preservation protocol required that the samples were processed as soon as possible after sampling. The ~10 mL of the original FNA mixture was pelleted for 5 min. and resuspended on ice in 2 mL NH_4_Cl lysis solution [59] to lyse contaminating erythrocytes. After a maximum of 5 min. with occasional gentle mixing on ice, and observation of the depletion of obvious erythrocytes, 1 mL of Accutase™ was added directly to the lysis buffer for a maximum of an additional 3 min., again with occasional gentle mixing and observation of the dissolution of obvious tissue clots in the solution. The sample volume was expanded with 6 mL ice cold RPMI buffer and the cells pelleted again. The pelleted cells were gently suspended in 200 µl PBS with 1% BSA preservation buffer [35] to which 1 mL of ice-cold methanol is slowly added with mixing. These preserved, sterilized, and de-identified samples were kept in the freezer or on ice packs for storage and transportation, followed by rehydration [35] and analysis by scRNA-seq.

### Single-cell RNA-sequencing

scRNA-seq was performed on single-cell suspensions using 10X Genomics Chromium to prepare cDNA sequencing libraries as described by Brady et al. [60]. Briefly, the 10X protocol (CG000136 • Rev D) instructs that the final sample should be in 200 ul of DPBS followed by the drop-wise addition of the MeOH. Following transport to the lab on ice, our samples were maintained at −80C until we were scheduled at the University of Utah High-Throughput Sequencing Core. The samples were allowed to equilibrate on ice for 10 min and then centrifuged at 400xg for 5 min at 4C. The supernatant was discarded, and the sample was resuspended in PBS +2% BSA supplemented with 0.2 U/ul RiboLock RNase inhibitor, filtered sequentially through a 70 and 40 um FlowMI tip filters to remove debris prior to submission to the HCI High Throughput Genomics Core facility.

The samples were processed using the Chromium Single Cell 3′ V3 Kit (10X Genomics, Cat. # 1,000,075) using whole cells fixed in 80% methanol. Single cells were diluted to a target of 1000 cell/μL in 1× PBS (whole cells) or 1× PBS +1.0% BSA +0.2 U/μL RiboLock RNase Inhibitor to generate GEM’s prepared at a target of 10,000 cells per sample. Barcoding, reverse transcription, and library preparation were performed according to manufacturer instructions. 10X Genomics generated cDNA libraries will be sequenced on NovaSeq 6000 instruments using 150 cycle paired-end sequencing at a depth of 10K reads per cell. The scRNA-seq was performed at the High Throughput Genomics Core at Huntsman Cancer Institute of the University of Utah.

For analytical procedures, the 10X Genomics Cell Ranger Single Cell software pipeline [35] is deployed to produce alignments and counts, utilizing the prescribed default parameters. The genomic references used for alignment were the human (hg38), the H37Rv Mtb (NC_00096.3:1) and HIV-1 (NC_001802.1). For quality management and further analytical exploration, Seurat (4.1.0) was utilized. Doublets were identified with DoubletFinder, cells were excluded based on having less than 100 genes/features and an excess of 25% mitochondrial genes. Mitochondrial genes were filtered out but every cell that contained Mtb genes was retained. Dimensionality was reduced and scaled via SCTransformation (0.3.5) using the Gamma-Poisson generalized linear model (glmGamPoi, 1.4.0) methodology at default resolution or less. Automated categorization of cells was performed using SingleR (1.6.1). Statistics within Seurat pipelines were generated with FindAllMarkers or FindMarkers which utilizes a Wilcoxon rank sum test [61–72].

### Performance of experiments in accordance with relevant guidelines and regulations statement

Medical Review Advisory Council (MRC/Institutional Review Board equivalent) approval for the work described here was obtained by Dr.s Evelyn Lavu (decd), Rodney Itaki, Jacklyn Joseph and Louis R. Barrows from UPNG School of Medicine, MRAC #54–6-2. All experiments were conducted in adherence to the Declaration of Helsinki. Current experiments performed under the aegis of Dr. Jacklyn Joseph, Coordinator of Pathology Services, Port Moresby General Hospital. All experiments were performed after obtaining verbal patient prior informed consent. Verbal consent was obtained due to the patients often being illiterate in English. All experiments were done in accordance with relevant guidelines and regulations. Approved protocols allowed up to 3 passed of fine needle aspiration using a 22-gauge needle for sampling lymph nodes of 0.8 cm diameter or larger.

### Inclusion and ethics

We are seek diversity and inclusion in our research team and promote equity in contributions and recognition. We strive for scientific rigor and truth in our research endeavors.

## Supplementary Material

257421405.R1 - Supplementary Material.docx

## Data Availability

Sequence data that support the findings of this study have been deposited in the NCBI Gene Expression Omnibus; accession numbers: GSE281148 (https://www.ncbi.nlm.nih.gov/geo/query/acc.cgi?acc=GSE281148) and GSE281835 (https://www.ncbi.nlm.nih.gov/geo/query/acc.cgi?acc=GSE281835). The.sam files can be found in figshare: https://doi.org/10.6084/m9.figshare.29141777.
