# Supplementary material for: T cell populations are negatively correlated with natural killer and macrophage cell populations in aspirate samples of peripheral lymphadenopathies
Source: Virulence. 2026 Jan 28;17(1):2624191. doi: 10.1080/21505594.2026.2624191 (PMC12885396; doi:10.1080/21505594.2026.2624191)
Supplement: 257421405.R1 - Supplementary Material.docx [file KVIR_A_2624191_SM7515.docx]

**Supplemental Figures**

| **Clusters** | **FNAs** | | | | | | | | | | | | | | | | | | | | | | | |
| --- | --- | --- | --- | --- | --- | --- | --- | --- | --- | --- | --- | --- | --- | --- | --- | --- | --- | --- | --- | --- | --- | --- | --- | --- |
|  | fna0 | fna1 | fna10 | fna11 | fna12 | fna13 | fna14 | fna15 | fna16 | fna17 | fna18 | fna19 | fna2 | fna20 | fna21 | fna22 | fna24 | fna25 | fna27 | fna28 | fna3 | fna4 | fna6 | fna8 |
| T Cell |  |  |  | 584 |  |  | 2734 | 6346 |  | 6838 |  | 724 |  |  |  | 421 | 1754 | 1894 | 13398 | 412 |  |  |  |  |
| B Cell | 471 |  |  | 193 | 7 | 102 | 1936 | 463 | 1681 | 2780 | 711 | 240 |  |  | 1367 | 154 | 509 | 1591 | 7573 | 149 |  |  |  |  |
| Macrophage/Dendritic | 159 |  | 645 | 59 | 123 | 191 | 1210 | 1267 | 57 | 341 | 4840 | 536 |  | 2579 | 47 | 86 | 437 | 46 | 102 | 248 |  |  | 7 |  |
| Plasma Cell | 62 |  |  | 66 | 2 | 68 | 200 | 2879 | 207 | 413 | 637 | 17 |  | 303 | 110 | 271 | 93 | 21 | 1804 | 19 |  |  |  |  |
| NK Cell |  |  |  | 420 |  |  | 2103 | 2741 |  | 789 |  | 2070 |  |  |  | 199 | 449 | 488 | 3560 | 610 |  |  |  |  |
| TB sum | 114 | 23 | 21 | 152 | 50 | 103 | 15 | 33 | 12 | 20 | 3 | 0 | 34 | 696 | 229 | 1 | 0 | 0 | 0 | 1 | 67 | 28 | 152 | 16 |
| T cell/NK | 859 |  | 465 | 1004 | 57 | 717 | 4841 | 9087 | 5314 |  | 1966 |  |  | 303 | 674 |  |  |  |  |  |  |  | 1295 |  |
| B cell/Plasma cell |  |  | 50 |  |  |  | 2163 | 3342 |  |  |  |  |  | 305 | 1477 |  |  |  |  |  |  |  | 1258 |  |
| Unknown/RBCs |  |  |  |  |  | 1746 UNK/Trans. Inact. | 15329 RBC |  |  |  | 2032 RBC |  |  |  |  |  |  |  |  |  |  |  |  |  |
| Total Bar Codes Sum with Max unless empty | 1808 | 3925 | 1118 | 1466 | 231 | 2855 | 23626 | 13593 | 6929 | 11418 | 7695 | 5656 | 3925 | 3609 | 2212 | 1126 | 3173 | 4075 | 26804 | 1426 | 3708 | 14883 | 2710 | 13678 |

**Fig. S-1** Sum of 1^st^ and max by K means clustering of total cell counts to match distinguishing features with Lasso tool and barcodes when necessary, from Fig. 2. UNK = transcriptionally inactive cells.

Fig. S-2 **Upper)** Combined Data Set Resolution 0.1, SingleR Fine from Figure 4. **Lower)** Extracted FNAs from combined 10X Genomics/Seurat SingleR data set.

| **lusters** | **FNAs** | | | | | | | | | | | | | | | | | | | |
| --- | --- | --- | --- | --- | --- | --- | --- | --- | --- | --- | --- | --- | --- | --- | --- | --- | --- | --- | --- | --- |
|  | fna0 | fna6 | fna10 | fna11 | fna12 | fna13 | fna14 | fna15 | fna16 | fna17 | fna18 | fna19 | fna20 | fna21 | fna22 | fna24 | fna25 | fna27 | fna28 | Sum |
| 0 = T1 | 880 | 928 | 261 | 521 | 30 | 323 | 2336 | 3788 | 1046 | 6207 | 386 | 1783 | 144 | 510 | 473 | 1125 | 1811 | 13076 | 277 | 35905 |
| 1 = NK | 62 | 69 | 119 | 414 | 16 | 190 | 2131 | 3954 | 214 | 814 | 215 | 7680 | 58 | 176 | 110 | 792 | 417 | 2430 | 668 | 20529 |
| 2 = B1 | 449 | 847 | 14 | 177 | 9 | 67 | 1769 | 436 | 1458 | 2587 | 627 | 691 | 1 | 1300 | 142 | 503 | 1516 | 6650 | 134 | 19377 |
| 3 = TI | 10 | 86 | 33 | 0 | 0 | 1863 | 0 | 31 | 0 | 1 | 5464 | 1 | 663 | 166 | 0 | 2 | 0 | 0 | 0 | 8320 |
| 4 = MMD | 172 | 13 | 8 | 78 | 103 | 43 | 970 | 946 | 77 | 160 | 28 | 2810 | 26 | 21 | 105 | 154 | 46 | 469 | 252 | 6481 |
| 5 = MM | 15 | 1 | 591 | 1 | 40 | 182 | 2 | 357 | 3 | 9 | 6 | 43 | 2549 | 16 | 13 | 339 | 2 | 15 | 1 | 4185 |
| 6 = T2 | 0 | 0 | 0 | 28 | 3 | 0 | 10 | 148 | 3661 | 6 | 0 | 19 | 0 | 0 | 2 | 0 | 0 | 1 | 0 | 3878 |
| 7 = P1 | 63 | 23 | 7 | 28 | 0 | 18 | 36 | 462 | 71 | 347 | 357 | 5 | 2 | 42 | 95 | 17 | 16 | 1558 | 0 | 3147 |
| 8 = P2 | 15 | 7 | 5 | 26 | 0 | 14 | 31 | 1020 | 30 | 186 | 158 | 10 | 1 | 16 | 60 | 25 | 10 | 1081 | 0 | 2695 |
| 9 = BMG | 17 | 192 | 21 | 19 | 6 | 28 | 66 | 863 | 74 | 573 | 5 | 23 | 17 | 63 | 16 | 56 | 55 | 143 | 3 | 2240 |
| 10 = P3 | 7 | 0 | 4 | 7 | 0 | 8 | 7 | 589 | 2 | 21 | 63 | 1 | 0 | 10 | 22 | 22 | 7 | 115 | 0 | 885 |
| 11 = P4 | 3 | 1 | 3 | 2 | 0 | 3 | 8 | 240 | 14 | 43 | 44 | 3 | 0 | 2 | 18 | 7 | 1 | 183 | 0 | 575 |
| 12 = TB | 10 | 90 | 9 | 2 | 12 | 3 | 12 | 0 | 0 | 0 | 0 | 4 | 76 | 12 | 0 | 0 | 0 | 0 | 2 | 232 |
| 13 = P5 | 0 | 0 | 0 | 0 | 0 | 0 | 0 | 213 | 0 | 0 | 0 | 0 | 0 | 0 | 0 | 0 | 0 | 0 | 0 | 213 |
| 14 = SE | 0 | 0 | 0 | 0 | 0 | 0 | 0 | 0 | 0 | 0 | 0 | 0 | 0 | 0 | 0 | 0 | 25 | 0 | 0 | 25 |
| **Sum** | 1703 | 2257 | 1075 | 1303 | 219 | 2742 | 7378 | 13047 | 6650 | 10954 | 7353 | 13073 | 3537 | 2334 | 1056 | 3042 | 3906 | 25721 | 1337 | 108687 |

Fig. S-3 Total cell counts for 15 clusters in Combined Data Set shown in Fig. 6 using Seurat UMAP clustering in figure 4A and S-2.

Fig. S-4A FNA cell composition trends calculated from extracted combined 10X Genomics/Seurat SingleR data set cluster cell number percentages/100, from Fig. 6A (Top Left). Data confirm results in Fig. 3.

Fig. S-4B FNA cell composition trends calculated from extracted combined Azimuth Cell reference-based mapping pipeline data set cluster cell number percentages/100, from Fig. 6A (Top Left). Data confirm results in Fig. 3.


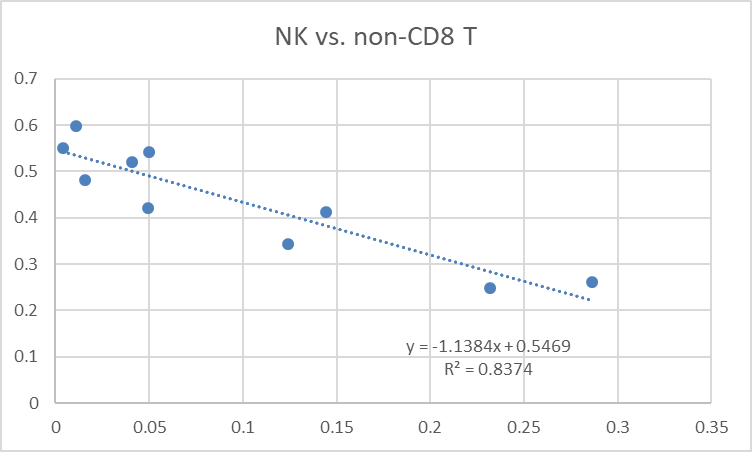


| NK% | % non-CD8**^+^** Ts | FNA |
| --- | --- | --- |
| 0.144282425 | 0.412893323 | fna11 |
| 0.124152887 | 0.344131201 | fna14 |
| 0.049973174 | 0.5423469 | fna15 |
| 0.004016798 | 0.551031587 | fna17 |
| 0.232004896 | 0.248298019 | fna19 |
| 0.049242424 | 0.420454545 | fna22 |
| 0.041091387 | 0.520381328 | fna24 |
| 0.015873016 | 0.480542755 | fna25 |
| 0.010886046 | 0.597954978 | fna27 |
| 0.286462229 | 0.260284218 | fna28 |

Fig. S-4C FNA cell composition trends calculated from extracted combined Azimuth cell reference-based mapping pipeline data set cluster cell number percentages/100, from Fig. 6A (Top Left). Data suggest results in Fig. 3 might be due to specific T cell subsets.


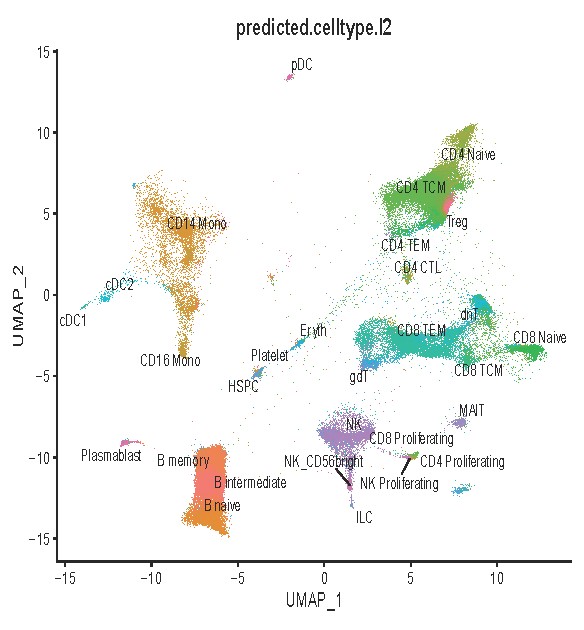


| Azimuth.l2 | fna0 | fna6 | fna10 | fna11 | fna12 | fna13 | fna14 | fna15 | fna16 | fna17 | fna18 | fna19 | fna20 | fna21 | fna22 | fna24 | fna25 | fna27 | fna28 |
| --- | --- | --- | --- | --- | --- | --- | --- | --- | --- | --- | --- | --- | --- | --- | --- | --- | --- | --- | --- |
| B intermediate | 225 | 502 | 95 | 100 | 2 | 56 | 926 | 255 | 364 | 1437 | 515 | 418 | 0 | 762 | 77 | 358 | 632 | 3123 | 47 |
| B memory | 45 | 153 | 173 | 25 | 2 | 5 | 270 | 316 | 109 | 744 | 207 | 251 | 2 | 65 | 13 | 88 | 156 | 3252 | 32 |
| B naïve | 196 | 64 | 2 | 55 | 5 | 11 | 501 | 117 | 1022 | 701 | 48 | 47 | 0 | 496 | 59 | 61 | 764 | 862 | 55 |
| CD14 Mono | 26 | 1 | 4670 | 50 | 109 | 159 | 606 | 407 | 2 | 25 | 4 | 1004 | 1701 | 13 | 67 | 381 | 10 | 33 | 216 |
| CD16 Mono | 2 | 0 | 2 | 5 | 9 | 4 | 219 | 63 | 14 | 13 | 1 | 362 | 2 | 3 | 23 | 4 | 4 | 24 | 29 |
| CD4 CTL | 1 | 0 | 22 | 0 | 0 | 0 | 64 | 0 | 0 | 0 | 0 | 291 | 1 | 0 | 0 | 1 | 2 | 1 | 2 |
| CD4 Naïve | 98 | 40 | 4 | 57 | 2 | 0 | 155 | 79 | 1724 | 530 | 13 | 129 | 0 | 3 | 107 | 10 | 132 | 498 | 17 |
| CD4 Proliferating | 0 | 0 | 5 | 10 | 3 | 8 | 10 | 134 | 21 | 209 | 1 | 12 | 6 | 21 | 4 | 19 | 12 | 39 | 0 |
| CD4 TCM | 590 | 1102 | 10162 | 373 | 45 | 522 | 1804 | 5023 | 1398 | 4442 | 1659 | 1819 | 490 | 387 | 233 | 907 | 1170 | 11252 | 210 |
| CD4 TEM | 3 | 22 | 1628 | 1 | 0 | 11 | 50 | 17 | 4 | 22 | 108 | 57 | 8 | 2 | 1 | 25 | 10 | 438 | 26 |
| CD8 Naïve | 111 | 20 | 15 | 60 | 1 | 2 | 128 | 95 | 1299 | 870 | 49 | 50 | 1 | 2 | 72 | 22 | 159 | 409 | 9 |
| CD8 Proliferating | 0 | 0 | 2 | 1 | 0 | 0 | 0 | 8 | 0 | 5 | 1 | 2 | 0 | 0 | 0 | 2 | 0 | 2 | 1 |
| CD8 TCM | 7 | 25 | 443 | 13 | 2 | 10 | 68 | 95 | 48 | 107 | 54 | 100 | 5 | 5 | 11 | 54 | 22 | 199 | 12 |
| CD8 TEM | 20 | 39 | 491 | 173 | 3 | 59 | 835 | 801 | 92 | 374 | 483 | 3430 | 37 | 42 | 37 | 235 | 159 | 1124 | 177 |
| cDC1 | 0 | 0 | 1 | 2 | 1 | 2 | 0 | 14 | 8 | 38 | 1 | 4 | 0 | 0 | 1 | 5 | 3 | 2 | 0 |
| cDC2 | 1 | 1 | 1 | 2 | 2 | 3 | 19 | 9 | 12 | 13 | 13 | 131 | 1 | 7 | 3 | 11 | 4 | 50 | 6 |
| dnT | 159 | 151 | 718 | 70 | 4 | 151 | 266 | 1707 | 212 | 446 | 450 | 110 | 62 | 330 | 61 | 527 | 274 | 1821 | 41 |
| Eryth | 81 | 38 | 24170 | 23 | 5 | 126 | 99 | 42 | 18 | 111 | 1689 | 880 | 736 | 68 | 49 | 3 | 29 | 349 | 1 |
| gdT | 1 | 2 | 13 | 4 | 1 | 1 | 66 | 4 | 17 | 12 | 6 | 752 | 1 | 0 | 2 | 11 | 7 | 20 | 31 |
| HSPC | 26 | 52 | 19503 | 1 | 5 | 1365 | 9 | 312 | 11 | 33 | 950 | 13 | 446 | 28 | 9 | 5 | 2 | 34 | 1 |
| ILC | 9 | 8 | 200 | 3 | 0 | 2 | 4 | 13 | 2 | 20 | 17 | 2 | 0 | 0 | 0 | 2 | 2 | 34 | 0 |
| MAIT | 2 | 0 | 34 | 17 | 7 | 23 | 165 | 73 | 9 | 3 | 22 | 78 | 13 | 0 | 5 | 33 | 4 | 5 | 17 |
| NK | 15 | 5 | 70 | 184 | 6 | 74 | 892 | 483 | 13 | 31 | 92 | 3022 | 20 | 7 | 43 | 101 | 58 | 200 | 380 |
| NK Proliferating | 1 | 1 | 69 | 0 | 1 | 14 | 10 | 90 | 1 | 4 | 1 | 9 | 18 | 2 | 4 | 24 | 3 | 4 | 0 |
| NK_CD56bright | 5 | 0 | 3 | 4 | 0 | 3 | 14 | 79 | 7 | 9 | 4 | 2 | 0 | 2 | 5 | 0 | 1 | 76 | 3 |
| pDC | 0 | 1 | 6 | 1 | 0 | 11 | 2 | 230 | 14 | 4 | 0 | 5 | 1 | 3 | 5 | 0 | 2 | 4 | 3 |
| Plasmablast | 28 | 12 | 153 | 47 | 0 | 40 | 74 | 2357 | 73 | 396 | 153 | 4 | 3 | 45 | 129 | 66 | 17 | 589 | 0 |
| Platelet | 1 | 0 | 2291 | 2 | 1 | 7 | 2 | 125 | 1 | 0 | 0 | 15 | 200 | 1 | 0 | 6 | 0 | 0 | 0 |
| Treg | 50 | 22 | 4526 | 20 | 3 | 73 | 120 | 99 | 155 | 355 | 812 | 74 | 34 | 40 | 36 | 81 | 268 | 1277 | 21 |

Fig. S-5 **Top)** Combined Azimuth cell reference-based mapping pipeline data set at Resolution 0.l2. **Bottom)** Combined Azimuth cell reference-based mapping pipeline data by FNA extracted from combined data set (data repeated from Fig. 5B).

| Sample | Sex | TB+ Con. | Year |
| --- | --- | --- | --- |
| FNA0 | M | Y | 2019 |
| FNA1 | M | ND | 2022 |
| FNA2 | M | ND | 2022 |
| FNA3 | M | ND | 2022 |
| FNA4 | F | Y | 2022 |
| FNA6 | M | Y | 2022 |
| FNA8 | M | ND | 2022 |
| FNA10 | M | Y | 2023 |
| FNA11 | F | ND | 2023 |
| FNA12 | F | ND | 2023 |
| FNA13 | M | Y | 2023 |
| FNA14 | F | ND | 2023 |
| FNA15 | F | ND | 2023 |
| FNA16 | F | ND | 2023 |
| FNA17 | F | N/RC | 2023 |
| FNA18 | F | Y | 2023 |
| FNA19 | M | N | 2023 |
| FNA20 | F | Y/RC | 2023 |
| FNA21 | F | Y/RC | 2023 |
| FNA22 | F | Y | 2023 |
| FNA24 | M | Y | 2023 |
| FNA25 | M | M | 2023 |
| FNA27 | M | ND | 2023 |
| FNA28 | M | ND | 2023 |

Table S 1 Patient/Sample data; TB confirmation by POMGH TB Clinic Pathology Lab using ZN staining and/or GeneXpert molecular analysis. Y=yes, N=no, RC=recurrent.
